# MFAP2, upregulated by m1A methylation, promotes colorectal cancer invasiveness via CLK3

**DOI:** 10.1002/cam4.5561

**Published:** 2022-12-30

**Authors:** Meng Xue, Shuyi Mi, Zizhen Zhang, Hao Wang, Wenwen Chen, Wei Wei, Guochun Lou

**Affiliations:** ^1^ Department of Gastroenterology The Second Affiliated Hospital of Zhejiang University School of Medicine Hangzhou China; ^2^ Institute of Gastroenterology, Zhejiang University Hangzhou China; ^3^ Department of Gastrointestinal Oncology, Key Laboratory of Carcinogenesis and Translational Research (Ministry of Education/Beijing) Peking University Cancer Hospital and Institute Beijing China

**Keywords:** colorectal cancer, invasiveness, MFAP2, N1‐methyladenosine

## Abstract

**Background:**

Distant metastasis is the main cause of mortality in colorectal cancer (CRC) patients. N1‐methyladenosine (m1A) is a type of epitranscriptome modification. While its regulatory effect on mRNA and its role in CRC metastasis remain unclear.

**Methods:**

The m1A methylation profile of mRNAs in CRC was revealed by m1A methylated RNA immunoprecipitation sequencing. The expression of MFAP2 in tumor tissues was measured by immunohistochemistry and then correlated with the clinical characteristics and prognosis of CRC patients. The role of MFAP2 in the invasiveness of CRC cells was evaluated by transwell assays and peritoneal metastatic model in nude mice. The downstream targets of MFAP2 was screened by mass spectrometry analysis. Then the role of MFAP2‐CLK3 signaling axis was verified by cotransfecting MFAP2 siRNA and CLK3 plasmid in CRC cells.

**Results:**

Microfibril associated protein 2 (MFAP2) mRNA was overexpressed and m1A‐hypermethylated in CRC. High expression of MFAP2 was closely related to lymph node metastasis and distant metastasis, leading to poor prognosis in patients with CRC. In vivo and in vitro studies showed that silencing of MFAP2 inhibited the migration, invasion and metastasis of CRC cells. CDC Like Kinase 3 (CLK3) was a potential downstream target of MFAP2. Further studies showed that MFAP2 depletion might induce autophagic degradation of CLK3, and the role of MFAP2 in the invasiveness of CRC cells was dependent on CLK3.

**Conclusions:**

Our results uncover a newly identified MFAP2‐CLK3 signaling axis, which is a potential therapeutic target for CRC metastasis.

## Graphical abstract

(1) MFAP2 mRNA was overexpressed and m1A‐hypermethylated in CRC. (2) MFAP2 promotes the metastasis of CRC via blocking the autophagic degradation of CLK3.

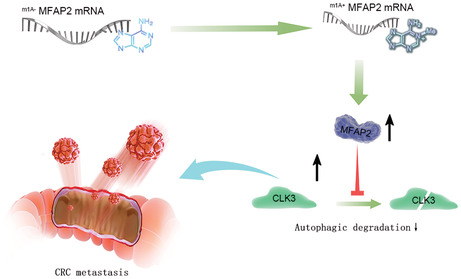

## 1 INTRODUCTION

Colorectal cancer (CRC) is the third most common cancer globally, and nearly one million people die from CRC every year worldwide.^1^ Over one‐fifth of CRC patients have distant organ metastasis at initial diagnosis, greatly impairing therapeutic effects.^2^ Although treatment strategies have made great progress in the past decades, the overall 5‐year survival rate of CRC patients with distant metastasis is still less than 15%.^3^ Clarifying the underlying mechanisms by which CRC metastasizes to distant organs is critical for the exploration of therapeutic targets.

Metastasis is accompanied by a series of genetic and epigenetic molecular events.^4^ Post‐transcriptional modification at the RNA level, known as epitranscriptome, has gained increasing attention because of its extensive role in tumor progression.^5^ N1‐methyladenosine (m1A) is a RNA methylation modification. Although the prevalence of m1A is lower than that of m6A, its effects on the structure and function of RNA are stronger in comparison to that of m6A, due to the presence of an additional positive charge in the modified nitrogen.^6^ A previous study analyzed data from The Cancer Genome Atlas (TCGA) and found that various m1A transmethylases and demethylases were correlated with CRC prognosis.^7^ Our previous investigation suggested that the profile of m1A modifications in lncRNAs, and several m1A‐modified lncRNAs were involved in CRC prognosis.^8^ However, the information available on m1A modification characteristics of mRNA in CRC is limited.

This study screened m1A‐dysregulated mRNAs in CRC and identified that microfibril associated protein 2 (MFAP2), a member of the MFAP family, had upregulated mRNA expression and m1A level. Furthermore, the role of MFAP2 in CRC invasion and metastasis were investigated, and its downstream molecular mechanisms were explored. These findings could provide insights for the identification of new therapeutic targets in CRC metastasis.

## 2 MATERIALS AND METHODS

### 2.1 Cell culture

HCT116 and RKO cells were purchased from American Type Culture Collection (ATCC), and cultured in McCoy's 5A and EMEM, respectively. The medium was supplemented with 10% fetal bovine serum (FBS; PAA Laboratories), and the cells were incubated at 5% CO_2_, 37°C and 95% humidity. All experiments were performed with mycoplasma‐free cells.

### 2.2 siRNAs and plasmids

ALKBH1/MFAP2/CLK3 siRNAs were synthesized by GenePharma and the sequences of the sense strand were 5′‐GUGAUCAAAUCUCAGCUAATT‐3′, 5′‐GCAGCAAG UCCAACAGGAATT‐3′, and 5′‐GAUGAUGGAGAAGAUC CUATT‐3′, respectively. The coding sequences of ALKBH1 (NM_006020)/MFAP2(NM_002403)/CLK3 (NM_003992) were amplified by reverse transcription‐polymerase chain reaction (RT‐PCR), ligated into the pGM‐T vector (Tiangen) and then subcloned into the XhoI/BamHI enzymatic sites of the pGMLV expression vector to generate CLK3 overexpression plasmids. All constructs were verified using DNA sequencing.

### 2.3 Cell transfection

For the loss‐of‐function assay, ALKBH1/MFAP2/CLK3 siRNAs were transfected into HCT116 and RKO cells using Lipofectamine RNAiMAX (Invitrogen), following the manufacturer's instructions. For gain‐of‐function assays in HCT116 and RKO cells, ALKBH1/MFAP2/CLK3 plasmids was transfected with PolyJet (SignaGen Laboratories), following the manufacturer's instructions.

### 2.4 Clinical sample preparation

Human ethics clearance was obtained from the Human Research Ethics Committee of the Second Affiliated Hospital, School of Medicine, Zhejiang University. For m1A methylated RNA immunoprecipitation (MeRIP) sequencing, three CRC patients provided informed consent before surgery, and CRC and adjacent normal fresh tissues from isolated specimens were collected. For immunohistochemistry (IHC), three cohorts including a total of 175 pairs (tumor and adjacent non‐tumorous tissues) of paraffin‐embedded specimens, were collected from the archive cabinet of the pathology department (cohort 1:90 pairs, cohort 2:28 pairs, and cohort 3:57 pairs). Accordingly, three tissue arrays were made in collaboration with the Outdo Biotechnology Company. For m1A methylation assay of MFAP2 mRNA in clinical samples, RNAs from tumor tissues of 27 CRC patients were collected.

### 2.5 MeRIP sequencing

Total RNAs were extracted from CRC and adjacent normal tissues using TRIzol reagent (#15596026; Invitrogen) and then sent to Cloud‐Seq Biotech for m1A RNA immunoprecipitation (RIP) sequencing. RNA quality assessment, library construction, and rough handling of raw data were performed as previously described.^8^ Peaks with *p* value <0.05, *q* value <0.1, and the FPKM in either CRC or adjacent normal tissues >1 were screened out. If more than one m1A peak was acquired for a single transcript, only the one that changed the most was selected for further analysis.

### 2.6 Quantitative real‐time PCR (qPCR)

After reverse transcription of the RNAs to cDNAs using HiScript Reverse Transcriptase (R101‐01, Vazyme), ChamQ Universal SYBR qPCR Master Mix (Q711‐02, Vazyme) was used for amplification using the primers listed in Table S1 (ACTB was used as an internal control). The reaction was performed on a LightCycler 480 Instrument (Roche), and the 2^−ΔΔCT^ method was used to calculate relative expression.

### 2.7 m1A MeRIP‐qPCR

One‐tenth volume of RNA was reserved as input. The remaining RNA was incubated with m1A antibody in RIP buffer (10 mM Tris HCl, 150 mM NaCl, 0.1% NP‐40, and 0.2% RNase inhibitor) overnight at 4°C. The RNA‐antibody complex was added into Protein A/G beads, prewashed with RIP buffer, and gently rotated overnight at 4°C. After the sediment was washed with RIP buffer, TRIzol, and chloroform were added to elute RNA from the beads. Then glycogen and isopropanol were added to the supernatant and incubated for 4 h at 4°C. After washing with 75% ethanol, the pellet was resuspended in RNase‐free water for further use. The precipitated RNAs and input RNAs were reverse‐transcribed and measured by qPCR. Relative enrichment of m1A^+^ mRNA was calculated as the 2^−ΔCt^ of m1A antibody precipitation relative to the input sample.^9^

### 2.8 IHC

Tissue array slides were baked in an oven, soaked in xylene, washed with alcohol, followed by antigen retrieval. Endogenous peroxidase and antibody nonspecific binding were blocked with 3% H_2_O_2_ and 10% goat serum, respectively. The slides were then incubated with MFAP2 primary antibody overnight at 4°C, followed by incubation with HRP‐labeled secondary antibody. The slides were stained with DAB and counterstained with hematoxylin. Staining intensity was categorized as follows: Zero (negative), one (weakly positive), two (medium positive), and three (strongly positive) (Figure S1). The percentage of positive cells was categorized as 0 (0%–5%), 1 (6%–50%), 2 (51%–75%) and 3 (76%–100%). Expression level = staining intensity × % of positive cells. The expression of MFAP2 protein was divided into low (≤3) and high (≥4) expression groups.^10^

### 2.9 Western blot

Total cellular proteins were lysed using RIPA buffer (Beyotime), loaded on an SDS‐PAGE gel, and transferred to PVDF membranes (Millipore). Primary antibodies against ALKBH1 (Abcam, ab126596, 1:1000), MFAP2 (Abclonal, A10230, 1:1000), BRD9 (CST, 58906 S, 1:1000), MPZL1 (CST, 9893 S, 1:1000), RIN1 (Abclonal, A13791, 1:1000), TMED9 (Abclonal, A3442, 1:1000), TRA2A (Absin, abs101809, 1:1000), CTSB (Abclonal, A19005, 1:1000), CMTM6 (Absin, abs148622, 1:1000), CLK3 (Absin, abs133041, 1:1000), and the internal control GAPDH (Diagbio, db106, 1:1000, Hangzhou, China) were used. After incubation with HRP‐linked rabbit secondary antibodies (Diagbio, db10002, 1:2500) and development with ECL substrate, the blots were visualized by chemiluminescence using the Azure 600 System (Azure Biosystems).

### 2.10 Mass Spectrometry (MS) analysis

The protein suspension samples were digested with trypsin (Promega) overnight at 37°C. The resulting peptides were labeled using the TMT reagent (Thermo Fisher Scientific) and then sent to LC Sciences for MS analysis. Briefly, reversed‐phase chromatography was performed to fractionate TMT‐labeled peptides. These fractionated peptide mixtures were loaded onto a C18‐reversed phase analytical column and separated using formic acid mixed with acetonitrile. LC–MS/MS analysis was performed on a Q Exactive Plus mass spectrometer (Thermo Fisher Scientific) in positive ion mode. The MS data were acquired by choosing the most abundant precursor ions from the survey scan. The output raw files were processed using the MASCOT engine (Matrix Science; version 2.6) embedded in Proteome Discoverer 2.2. A precursor mass tolerance of 10 ppm and a 0.05 Da tolerance for the fragments were specified. Proteins with a fold change >1.3 and a *p* value <0.1 were considered to be differentially expressed.

### 2.11 Transwell assay

To evaluate cell migration and invasion, transwell assays were performed using transwell filters (pore size, 8 μm; BD Biosciences) with or without matrigel (BD Biosciences). Transfected HCT116 and RKO cells were seeded into the upper chambers with 1% serum medium, while the lower chambers were supplemented with 15% medium. After 24 h, the non‐migrated or non‐invaded cells were carefully removed with cotton swabs, and the cells passing through the membrane were fixed with 4% paraformaldehyde and then stained with 0.1% crystal violet. Subsequently, cells in five random fields were counted under a microscope.

### 2.12 Xenograft metastatic model

Animal experiments were approved by the Animal Care and Use Committee of the Second Affiliated Hospital, School of Medicine, Zhejiang University. HCT116 cells were first transfected with GV493‐MFAP2‐shRNA lentivirus (NM_002403, shRNA sequence: 5′‐CTCCATACACAGGCCTTGCAA‐3′), and puromycin was used to establish MFAP2 stably silenced cell line. Next, PGMLV‐CMV‐luc‐PGK‐neo was transfected for luciferase detection. About 5–6 weeks old female BALB/c nude mice were purchased from Vital River. Resuspended solution (200 μl) containing 2 × 10^6^ MFAP2 stably silenced HCT116 cells was injected intraperitoneally. Six weeks later, the mice were intraperitoneally administered D‐luciferin (1.5 mg) and then imaged using IVIS (PerkinElmer IVIS Lumina XRMS Series III imaging system) to measure the luciferase signal intensity every week.^11^ When a marked difference in the luciferase signal intensity was observed between the two groups, the mice were euthanized.

### 2.13 Statistical analysis

SPSS software (version 21.0) was used for statistical analysis. The difference in MFAP2 expression between tumor and adjacent normal tissues was determined using the Mann–Whitney *U*‐test. Chi‐square test was used to correlate MFAP2 staining scores and clinicopathological characteristics. The overall survival probability was tested using the Kaplan–Meier method, and the difference between curves was evaluated using the log‐rank test. The difference in the rest was calculated using Student's *t*‐test. Statistical significance was set at *p* < 0.05.

## 3 RESULTS

### 3.1 m1A upregulated the expression of MFAP2 in CRC cells

**FIGURE 1:**
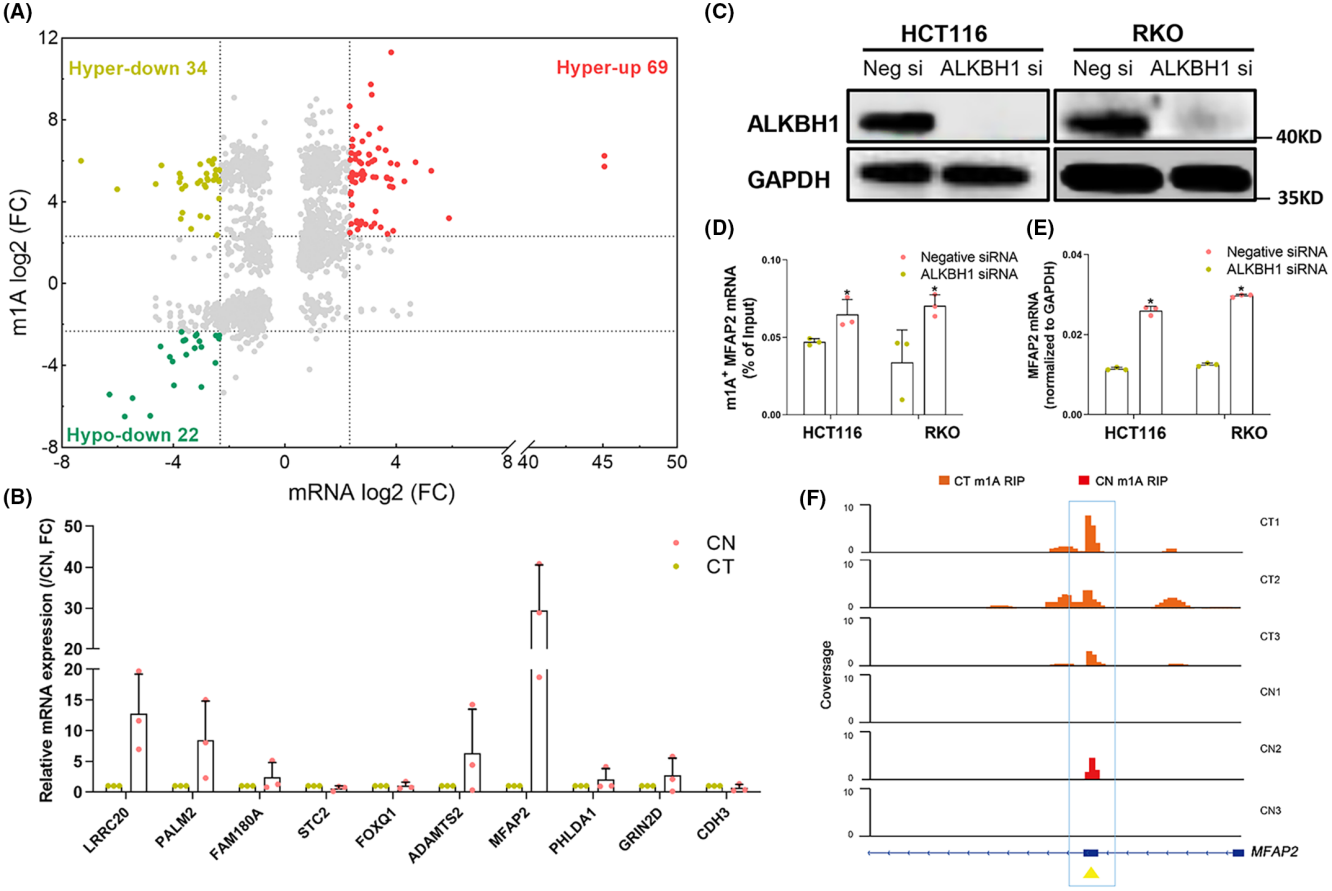
m1A upregulated the expression of MFAP2 in CRC cells. (A) Star plot shows the distribution of mRNAs with altered m1A peaks. The fold change of either expression or m1A methylation of mRNA <2 are labeled as gray dots, while the others are marked with dots of different colors (Hyper and Hypo indicate m1A methylation, up and down indicate mRNA expression) (*n* = 3). (B) qPCR validation of the genes with the FPKM values increased most markedly in CRC tissues (*n* = 3). (C) The transfection efficiency of ALKBH1 siRNA in HCT116 and RKO cells was confirmed by Western blot (*n* = 3). (D) Relative enrichment of ^m1A+^ MFAP2 mRNA in HCT116 and RKO cells after transfecting ALKBH1 siRNA (*n* = 3). (E) The expression of MFAP2 mRNA in HCT116 and RKO cells after transfecting ALKBH1 siRNA. (F) Visual m1A peak plot along the mRNA sequence of MFAP2 in colorectal tumor (CT) and adjacent normal tissues (CN). **p < 0.01, *p < 0.05; FC, fold change; Neg, Negative; si, siRNA; CT, colorectal tumor; CN, adjacent normal tissues; RIP, RNA immunoprecipitation.

Through MeRIP sequencing, the m1A methylation and expression profiles of mRNA in CRC were simultaneously screened. These sequence data have been submitted to the EMBL databases under accession number ERS13471606. To correlate m1A methylation status with mRNA expression, a cross analysis was performed. When setting fivefold as the alteration threshold, more mRNAs were upregulated along with the m1A modification (Figure 1A). Ten genes with the greatest increase in FPKM values in CRC tissues (Table S2) were selected for further qPCR validation, which showed that LRRC20 and MFAP2 were upregulated by over 10‐folds (Figure 1B).

m1A is regulated by the “writer” (TRMT10C/61B/6/61A), “eraser” (ALKBH1/3), and “reader” (YTHDF1/2/3 and YTHDC1). Among them, the eraser ALKBH1 is the regulator studied most, and often used as a tool to modify the m1A methylation status.^9^ So, we transfected ALKBH1 siRNA into HCT116 and RKO cells (Figure 1C), and then measured the m1A methylation levels of LRRC20 and MFAP2 mRNAs by m1A MeRIP‐qPCR. It showed that silencing ALKBH1 could upregulate ^m1A+^MFAP2 mRNA (Figure 1D), instead of ^m1A+^LRRC20 mRNA (Figure S2). Simultaneously, MFAP2 mRNA expression was upregulated (Figure 1E). After that, ALKBH1 plasmid was co‐transfected in ALKBH1 silenced HCT116 and RKO cells, and m1A MeRIP‐qPCR showed that reintroducing ALKBH1 in CRC cells would block the upregulation of ^m1A+^MFAP2 mRNA induced by ALKBH1 siRNA (Figure S3). Furthermore, the visual m1A peak plot also showed an accumulation of m1A modification along the mRNA sequence of MFAP2 in CRC when compared to adjacent normal tissues (Figure 1F).

### 3.2 MFAP2 was correlated with metastasis in CRC patients

To delineate the expression pattern of MFAP2 in CRC tissues, three tissue slides from a total of 175 CRC patients were stained using MFAP2 antibody. The demographic information of these patients were listed in Table S3. During the IHC assay, one point of CRC tissue and two points of adjacent normal tissues in cohort 1, and another one point of normal tissues in cohort 3 were detached from the slides. As a result, 171 paired samples were recruited to compare the expression of MFAP2 between tumor and tumor‐free tissues, while 174 CRC samples were used to correlate the expression of MFAP2 with clinical characteristics.

**FIGURE 2:**
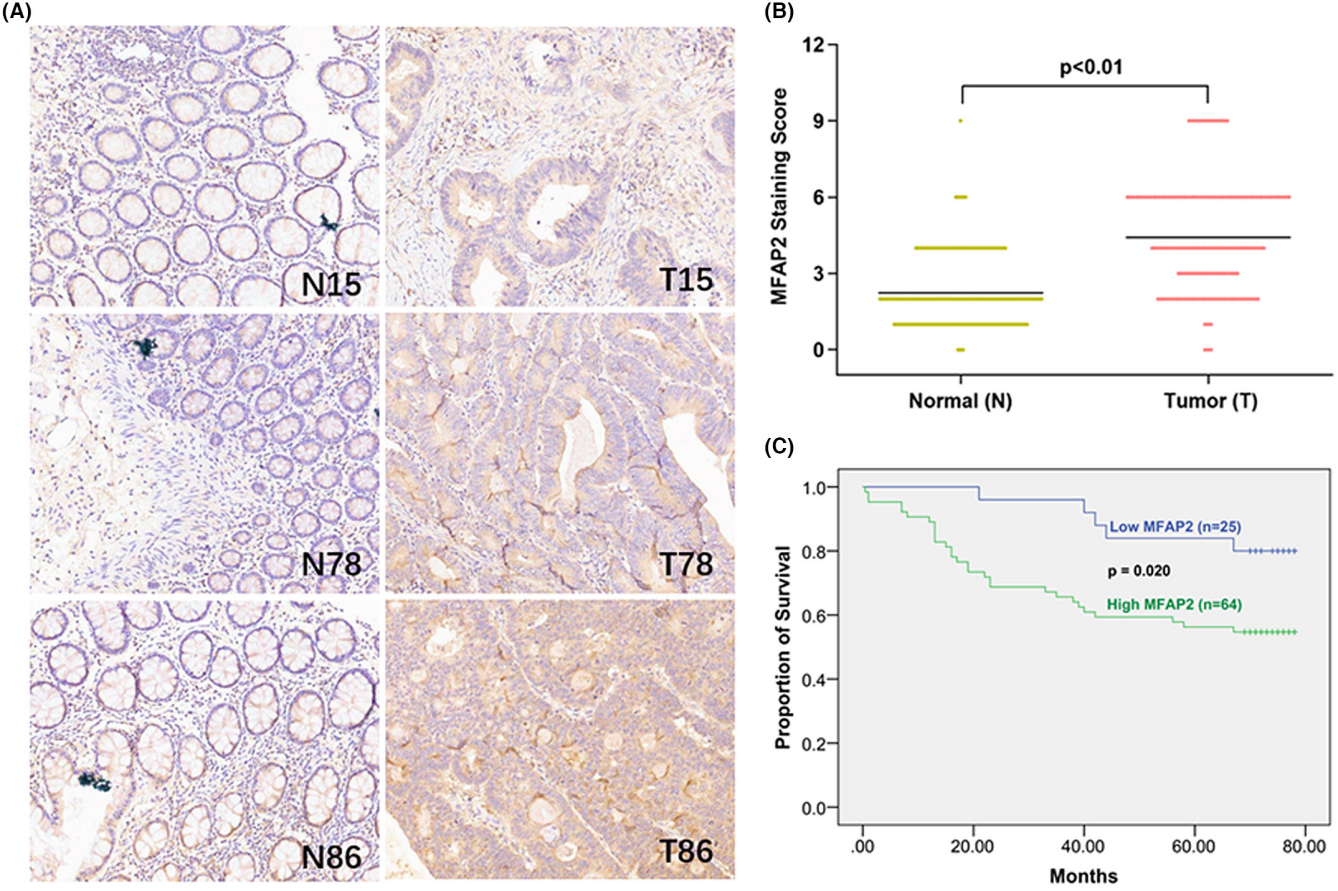
MFAP2 was correlated with metastasis and poor prognosis in CRC patients. (A) Representative immunohistochemistry images of MFAP2 protein expression in 3 paired CRC tissues and adjacent normal tissues. (B) The comparison of staining scores of MFAP2 between tumor and adjacent tumor free tissues from 171 CRC patients (*n* = 171). (C) Survival curves stratified by the expression level of MFAP2 were plotted based on the Kaplan–Meier analysis (*n* = 89). N, adjacent normal tissues; T, CRC tissues.

Representative immunostaining images were presented in Figure 2A. Statistical analysis showed that the expression of MFAP2 in CRC tissues was significantly higher than that in tumor‐free tissues (Figure 2B). Then the expression of MFAP2 in CRC tissues were correlated with the clinicopathological characteristics of the patients. High expression of MFAP2 in CRC tissues was significantly associated with lymph node metastasis (N0, 52/95 vs. N1/2/3, 58/79; *p* = 0.011), distant metastasis (M0, 87/145 vs. M1, 23/29; *p* = 0.049), and advanced AJCC stage (stage 1/2, 49/92 vs. stage 3/4, 61/82, *p* = 0.004) (Table 1).

**TABLE 1:** The correlation between the expression of MFAP2 and the clinicopathological characteristics in CRC

Clinical classification	Total number	MFAP2 expression		
		Low (number)	High (number)	p Value
Gender				
Male	91	29	62	0.159
Female	83	35	48	
Age (years)				
≥65	90	34	56	0.778
<65	84	30	54	
T stage				
T1 + T2	17	8	9	0.355
T3 + T4	157	56	101	
N stage				
N0 + N1	95	43	52	0.011*
N2	79	21	58	
M stage				
M0	145	58	87	0.049*
M1	29	6	23	
AJCC stage				
AJCC1/2	92	43	49	0.004**
AJCC3/4	82	21	61	

Abbreviations: AJCC, American Joint Committee on Cancer; N, lymph node; M, metastasis; T, tumor.

* *p* < 0.05

** *p* < 0.01.

As only cohort 1 had follow‐up information, survival analysis was performed based on 89 CRC tissue samples. The Kaplan–Meier analysis showed that CRC patients with high MFAP2 expression in tumor tissues had a lower overall survival rate than those with low MFAP2 expression (*p* = 0.020, Figure 2C).

In order to recognize the involvement of ^m1A+^MFAP2 in the clinical stages of CRC, the m1A methylation of MFAP2 mRNA in tumor tissues from 27 CRC patients was measured. The results showed that the MFAP2 m1A methylation level in CRC patients at N1‐2, M1, AJCC3 + 4 stages was higher than those patients at N0, M0, AJCC1 + 2 stages (Figure S4).

### 3.3 Depletion of MFAP2 inhibited the invasion and metastasis of CRC cells

**FIGURE 3:**
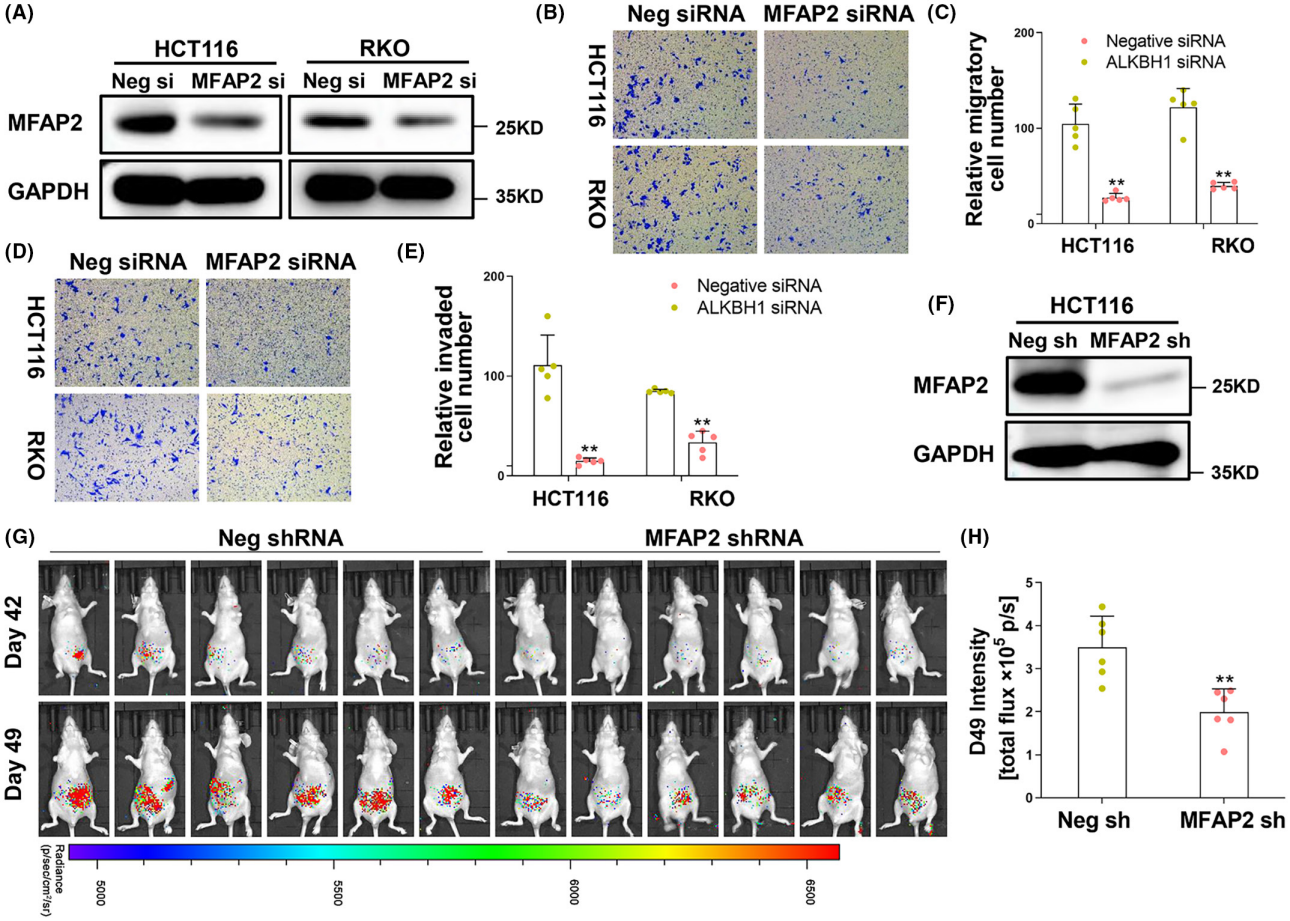
Depletion of MFAP2 inhibited the invasion and metastasis of CRC. (A) The transfection efficiency of MFAP2 siRNA in HCT116 and RKO cells was confirmed by Western blot. (B) After seeded in the upper transwell chamber and incubation for 24 h, HCT116 and RKO cells that migrated to the bottom of the membrane were stained with crystal violet. The representative images were captured under the microscope at 200×. (C) The average number of migratory cells was counted in five random fields (*n* = 5). (D) After seeded in the matrigel‐coated transwell chamber and incubation for 24 h, HCT116 and RKO cells that invaded to the bottom of the membrane were stained with crystal violet. The representative images were captured under the microscope at 200×. (E) The average number of invaded cells was counted in five random fields (*n* = 5). (F) The transfection efficiency of MFAP2 shRNA in HCT116 cells was measured by Western blot. (G) In vivo bioluminescence imaging of the mice injected with MFAP2 shRNA transfected HCT116 cells at the sixth and seventh week. (H) Quantitative analysis of bioluminescence imaging intensity on Day 49 (*n* = 6). **p < 0.01; Neg, Negative; si, siRNA; sh, shRNA.

To determine the role of MFAP2 in the invasiveness of CRC cells, MFAP2 siRNA was transfected in HCT116 and RKO cells (Figure 3A). Transwell assays with simple chambers or matrigel‐coated chambers were performed. Under the microscope, fewer cells passing through the membrane were observed (Figure 3B–E).

To further confirm the effect of MFAP2 on CRC in vivo, MFAP2 stably silenced HCT116 cells (Figure 3F) were injected into nude mice to establish a peritoneal metastasis model. In vivo luciferase bioluminescence imaging showed that luciferase signal intensity was weaker in MFAP2 silenced HCT116 cells (Figure 3G–H).

In addition, MFAP2 plasmid was co‐transfected in MFAP2 silenced HCT116 and RKO cells. Transwell assays showed that reintroducing MFAP2 in CRC cells would increase the migrated and invaded cells inhibited by MFAP2 siRNA (Figure S5). All the above in vivo and in vitro studies indicate that the invasiveness of CRC cells could be enhanced by MFAP2.

### 3.4 Suppression of MFAP2 induced the degradation of CLK3

**FIGURE 4:**
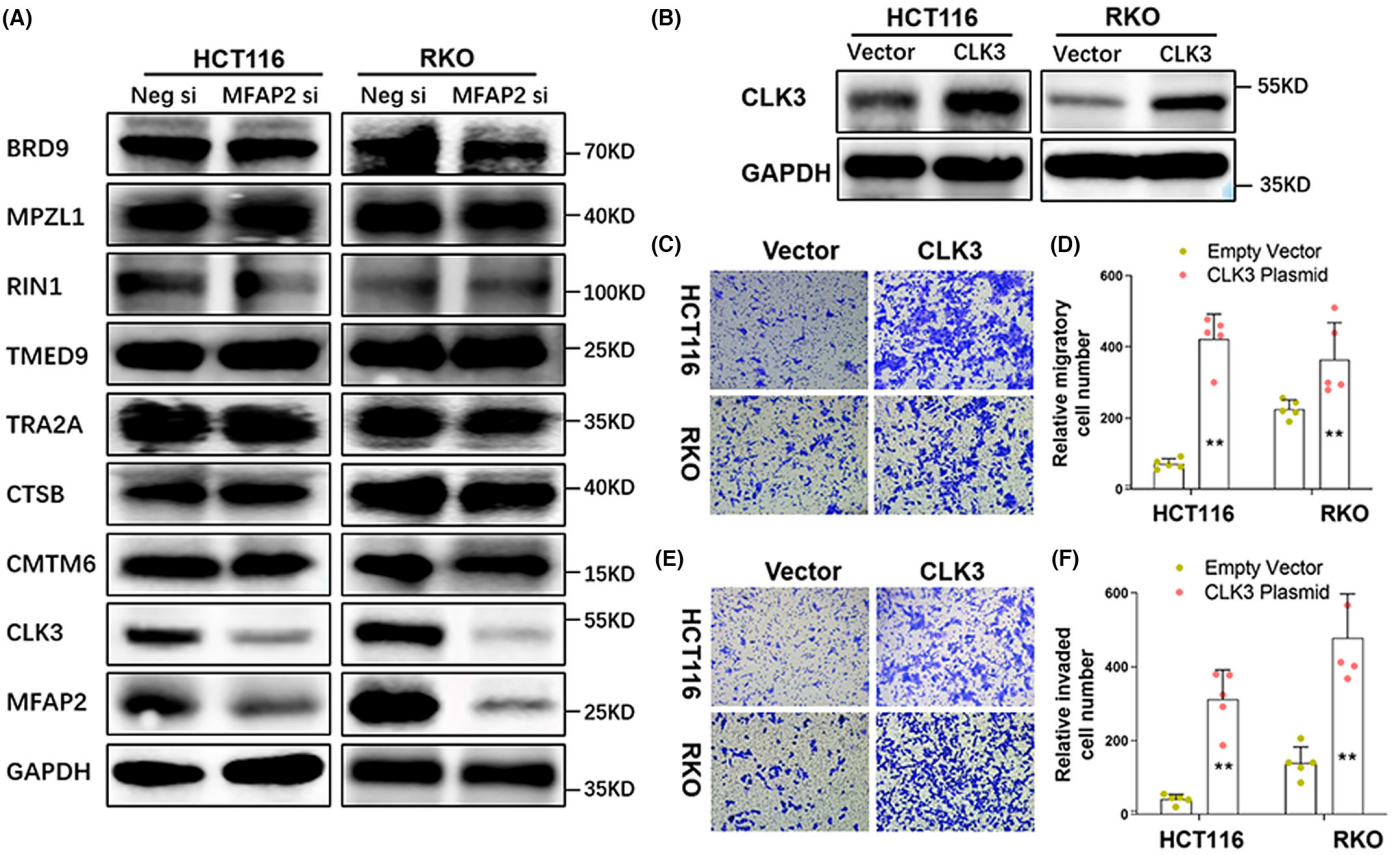
Suppression of MFAP2 inhibited the expression of proinvasive factor CLK3 in CRC cells. (A) Eight potential downstream targets of MFAP2 were measured by Western blot after MFAP2 was silenced in HCT116 and RKO cells. (B) The transfection efficiency of CLK3 ectopic plasmid in HCT116 and RKO cells was evaluated by Western blot. (C) After seeded in the upper transwell chamber and incubated for 24 h, CLK3 overexpressed HCT116 and RKO cells that migrated to the bottom of the membrane were stained with crystal violet. The representative images were captured under the microscope at 200×. (D) The average number of migratory cells was counted in five random fields (*n* = 5). (E) After seeded in the matrigel‐coated transwell chamber and incubated for 24 h, CLK3 overexpressed HCT116 and RKO cells that invaded to the bottom of the membrane were stained with crystal violet. The representative images were captured under the microscope at 200×. (F) The average number of invaded cells was counted in five random fields (*n* = 5). **p < 0.01; Neg, Negative; si, siRNA.

To elucidate the molecular mechanisms by which MFAP2 regulates the invasiveness of CRC cells. We measured the protein spectrum using MTM‐labeled LC–MS/MS after interfering MFAP2 in HCT116 cells, and a total of 77 proteins (30 down and 47 up) were filtered out (Table S4). We then searched for the role of the corresponding genes in cancer based on previous studies from PubMed, and eight downregulated oncogenes were selected as potential downstream targets (Table S5). Next, the protein expression of these eight genes was measured in MFAP2 silenced HCT116 and RKO cells. Western blotting showed that CLK3 protein expression was markedly reduced in both cell types after siRNA transfection (Figure 4A). However, CLK3 mRNA expression was not altered by MFAP2 siRNA (Figure S6).

The role of CLK3 in the invasiveness of CRC cells was then validated by transwell assays. CLK3 overexpressed HCT116 and RKO cells (Figure 4B) were seeded in uncoated or matrigel‐precoated chambers. Crystal violet staining of the lower membrane indicated that more CRC cells overexpressing CLK3 could climb through the micropores of the transwell chambers (Figure 4C–F). Besides, the expression of CLK3 was checked in surgical CRC samples from the GEO dataset of GSE131418 (includes 545 non‐metastatic and 73 metastatic CRC samples), showing that the relative mRNA expression of CLK3 in CRC at M1 stage was higher than that in CRC at M0 stage (Figure S7).

In order to observe whether CLK3 affect the expression of MFAP2, CLK3 siRNA was transfected in HCT116 and RKO cells, qPCR, and Western blot showed that neither mRNA expression nor protein expression of MFAP2 was altered (Figure S8). For loss‐of‐function assay, transwell migration assay was applied using CRC cells transfected with CLK3 siRNA, showing that CLK3 knockdown could inhibit CRC cell migration (Figure S9).

### 3.5 MFAP2 regulated the invasiveness of CRC cells through CLK3

**FIGURE 5:**
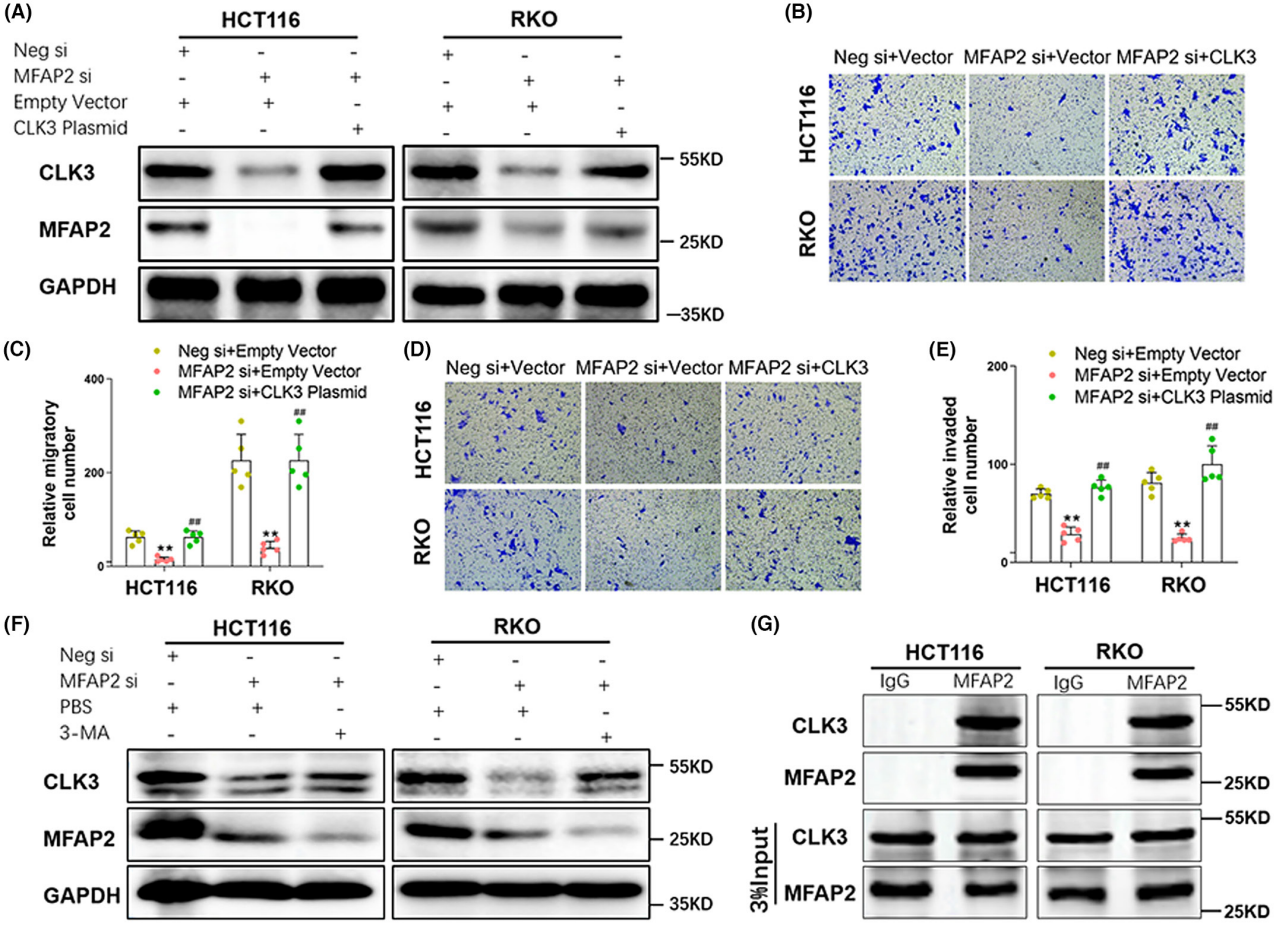
MFAP2 regulated the invasiveness of CRC cells through CLK3. (A) HCT116 and RKO cells were transfected with negative control siRNA or MFAP2 siRNA, together with empty vector or CLK3 ectopic plasmid. The transfection efficiency was assessed by Western blot. (B & C) After seeded in the transwell chamber for 24 h, HCT116 and RKO cells at the bottom of the membrane were stained with crystal violet. The representative images were captured under the microscope at 200×, and the average number of migratory cells was counted in five random fields (*n* = 5). (D & E) After seeded in the matrigel‐coated chamber for 24 h, HCT116 and RKO cells at the bottom of the membrane were stained with crystal violet. The representative images were captured under the microscope at 200×, and the average number of invaded cells was counted in five random fields (*n* = 3). (F) CLK3 protein was measured by Western blot in MFAP2 silenced CRC cells after 3‐MA treatment. (G) HCT116 and RKO cells were harvested and lysed. Immunoprecipitation analyses were carried out using IgG or MFAP2 antibodies. Three percent of the total input is shown. **p < 0.01 versus Neg si + Empty Vector; ^##^ *p* < 0.01 versus MFAP2 si + Empty Vector; Neg, Negative; si, siRNA.

To determine if the role of MFAP2 in CRC invasiveness was dependent on CLK3, a CLK3 ectopic plasmid was co‐transfected into MFAP2 silenced CRC cells. The co‐transfection efficiency was confirmed by western blot analysis (Figure 5A). Transwell migration and invasion assays showed that re‐expression of CLK3 significantly attenuated the repression of CRC cells moving to the lower chamber induced by MFAP2 depletion (Figure 5B–E).

Considering that autophagy and ubiquitination are two common mechanisms in protein degradation,^12^ we estimated the protein expression of CLK3 after adding the autophagic inhibitor 3‐MA (2.5 mM) or the ubiquitin proteasome inhibitor MG132 (5 μM) to MFAP2 silenced CRC cells. The blot results showed that addition of 3‐MA increased the protein level of CLK3 inhibited by MFAP2 silencing (Figure 5F), while MG132 failed (Figure S10). Immunoprecipitation with anti‐MFAP2 antibody revealed that MFAP2 was able to interact with CLK3 (Figure 5G), indicating that MFAP2 might bind with CLK3 and induce its degradation via autophagic pathway.

## 4 DISCUSSION

N1‐methyladenosine is a reversible type of RNA methylation located at the Watson–Crick base‐pairing interface.^13^ It was assumed to affect gene expression positively, evidenced by the fact that upregulated mRNAs often had an accumulated m1A modification.^6^ In addition, m1A alterations can promote translation efficiency.^14^ In our study, m1A hypermethylated transcripts had increased mRNA expression, whereas none of the m1A hypomethylated transcripts were upregulated at the mRNA level. Besides, knock‐down of the demethylase ALKBH1 increased MFAP2 mRNA expression levels and m1A methylation. Thus, the increased expression of MFAP2 in CRC clinical specimens could be explained by enhanced m1A modifications.

Similar to m6A modification, m1A is also regulated by transmethylases such as TRMT10C and demethylases such as ALKBH3.^15^ Yamato et al.^16^ found that ALKBH3 induced apoptotic resistance and vessel formation in pancreatic cancer. Another study showed that ALKBH3 inhibited tumorigenesis in an azoxymethane/dextran sodium sulfate (AOM/DSS) model.^17^ However, the role of m1A‐regulated transcripts in CRC remains unclear. This study explored the role of MFAP2 in the invasiveness of CRC cells and found that MFAP2 is a pro‐invasive factor, in vitro and in vivo. In addition, protein measurement of MFAP2 in CRC clinical specimens also verified its pro‐metastatic role.

MFAP2 is an extracellular matrix glycoprotein that plays important roles in microfibril assembly, elastinogenesis, and tissue homeostasis.^18^ Chen et al.^19^ found that MFAP2 promoted the invasion of melanoma cells through Wnt/β‐catenin activation. Another study showed that MFAP2 encouraged cell migration and invasion in gastric cancer by enhancing the PI3K‐Akt pathway.^20^ As to CRC, a recent study showed that LncRNA‐KCNQ1OT1 could promote the migration of SW480 and HCT116 cells, depending on MFAP2.^21^ However, the effect of MFAP2 on CRC invasion and its underlying mechanisms remain unclear. In this study, we comprehensively measured protein alterations after silencing MFAP2, and identified CLK3 as its downstream target.

CLK3 is a member of the CDC like splice factor kinase (CLK) family, displaying dual‐specificity kinase activity.^22^ Zhou et al.^23^ reported that CLK3 was involved in the regulation of purine metabolism in cholangiocarcinoma, and depletion of CLK3 blocks the invasion and metastasis of cholangiocarcinoma cells. Another study showed that CLK3 was a target of miR‐144, and overexpression of CLK3 could reverse the inhibited invasiveness of liver cancer cells by miR‐144.^24^ We observed that the migration and invasion capacities of CRC cells were induced by CLK3, and rescue assays confirmed that the role of MFAP2 in CRC was dependent on CLK3. The intracellular protein concentration is determined by the dynamic balance between synthesis and degradation. Autophagy and ubiquitination are the two most prevalent mechanisms in protein degradation,^25^ which can be explored by adding the autophagy inhibitor 3‐MA or proteasome inhibitor MG132.^26^ In this study, the detection of altered CLK3 after the addition of 3‐MA suggested the regulatory mechanisms of CLK3 protein by MFAP2.

This study has several limitations. First, the specific m1A modification site along MFAP2 mRNA is still unknown. Considering that adenosine methylation could block Watson‐Crick base pairing, thus affecting reverse transcription,^27^ the modification site could be confirmed by counting the mutation frequency in sequencing. Second, the underlying mechanisms by which MFAP2 regulates the autophagic degradation of CLK3 protein are also unclear. The kinase pathway^28^ involved in this process needs to be further investigated. Third, MFAP2 is a secretory protein. Whether MFAP2 exerts its functions through cell membrane receptors, or enters CRC cells needs to be further explored.

## 5 CONCLUSIONS

**FIGURE 6:**
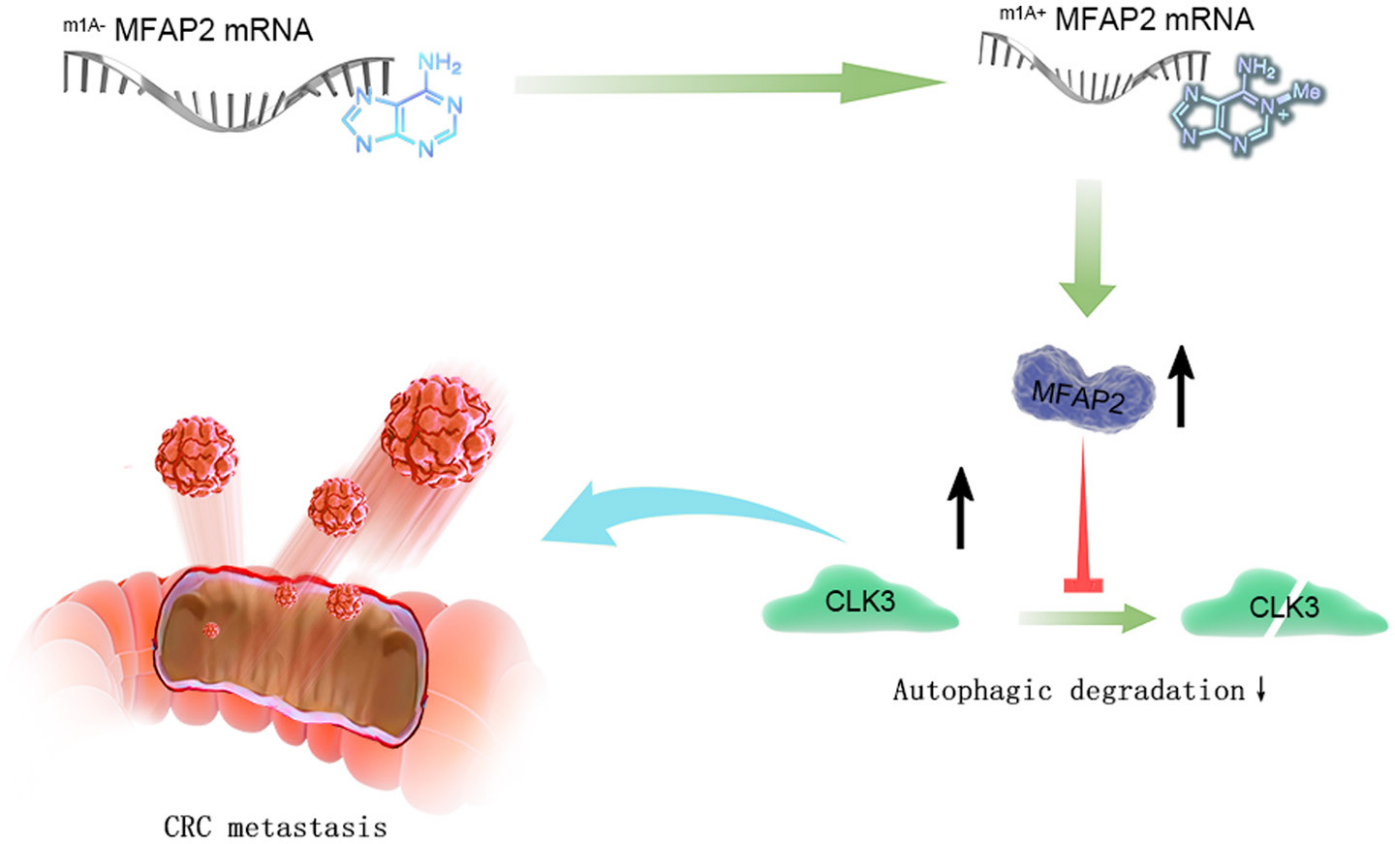
Working model of MFAP2 promoting CRC metastasis through CLK3. In CRC, m1A induces the expression of MFAP2 mRNA. Accumulated MFAP2 inhibits the autophagic degradation of CLK3, which will promote the metastasis of CRC.

In summary, we found that MFAP2 mRNA was upregulated in CRC cells owing to m1A methylation. Targeting MFAP2 could inhibit the invasion and metastasis of CRC, potentially through autophagic degradation of downstream CLK3 (Figure 6). We hope that this study provides a new therapeutic target for CRC metastasis.

## AUTHOR CONTRIBUTIONS

**Meng Xue:** Investigation (lead); writing – original draft (equal); writing – review and editing (equal). **Shuyi Mi:** Investigation (supporting); writing – original draft (equal); writing – review and editing (equal). **Zizhen Zhang:** Investigation (supporting). **Hao Wang:** Methodology (equal); resources (equal). **Wenwen Chen:** Software (equal). **Wei Wei:** Project administration (equal); supervision (supporting). **Guochun Lou:** Project administration (equal); supervision (lead).

## FUNDING INFORMATION

The present study was supported by the National Natural Science Foundation of China (82073229).

## CONFLICT OF INTEREST

The authors declare no conflict of interest.

## ETHICS APPROVAL STATEMENT

Human ethics approval was obtained from the Human Research Ethics Committee of the Second Affiliated Hospital, School of Medicine, Zhejiang University. Informed consent was obtained from all the participants. Animal experiments were approved by the Animal Care and Use Committee of the Second Affiliated Hospital, School of.

## PATIENT CONSENT STATEMENT

Three CRC patients provided informed consent before surgery. After that, CRC and adjacent normal fresh tissues from isolated specimens were collected.

## Supporting information


Table S1.

Table S2.

Table S3.

Table S4.

Table S5.

Figure S1–S10.

## Data Availability

All data included in this study are available upon request by contact with the corresponding author.
