# Supplementary material for: MFAP2, upregulated by m1A methylation, promotes colorectal cancer invasiveness via CLK3
Source: Cancer Med. 2022 Dec 30;12(7):8403–14. doi: 10.1002/cam4.5561 (PMC10134263; doi:10.1002/cam4.5561)
Supplement: Supplementary file 1 — Table S1. [file CAM4-12-8403-s005.docx]

**Supplementary Table S1. qPCR primers.**

| Gene Name | Forward Primer | Reverse Primer |
| --- | --- | --- |
| LRRC20 | GTGCAAGCTGGTCTCCTTTC | TTAAGCTCGTTGTTAGCCAGG |
| PALM2 | GACGAAAAAGGTGCTAGGCTAT | CGTCCGTCACTGTCTTCTCC |
| FAM180A | TGGAGCTGCTCTACGAGTTC | GCACTGTCCTTTCAAAGTCTTCT |
| STC2 | ACAGGTTCGGCTGCATAAGC | GAGGTCCACGTAGGGTTCG |
| FOXQ1 | CACGCAGCAAGCCATATACG | CGTTGAGCGAAAGGTTGTGG |
| ADAMTS2 | GTGCATGTGGTGTATCGCC | AGGACCTCGATGTTGTAGTCA |
| MFAP2 | TCCGCCGTGTGTACGTCATT | CTGGCCATCACGCCACATTT |
| PHLDA1 | GAAAAAGTGTTGCATCCTCACC | AGTCCACGGTCTTCATGTTGG |
| GRIN2D | GTGGGATAACCGGGATTACTCC | GAAGCGACCATAGCGGGAC |
| CDH3 | ACAATGGGGTGGTTGCTTACT | TGGCATCAAGGATCTCCACTA |
| CLK3 | TATGGACCTTCACGTTCTCGT | CGCTGCTACAAGACCTGGTG |
| ACTB | CATGTACGTTGCTATCCAGGC | CTCCTTAATGTCACGCACGAT |
