# Supplementary material for: MFAP2, upregulated by m1A methylation, promotes colorectal cancer invasiveness via CLK3
Source: Cancer Med. 2022 Dec 30;12(7):8403–14. doi: 10.1002/cam4.5561 (PMC10134263; doi:10.1002/cam4.5561)
Supplement: Supplementary file 2 — Table S2. [file CAM4-12-8403-s004.docx]

**Supplementary Table S2. Ten genes with the FPKM values increased the greatest in CRC tissues.**

| Gene Name | CT/CN m1A log2 (FC) | CT/CN mRNA log2 (FC) |
| --- | --- | --- |
| LRRC20 | 6.25 | 45.11 |
| PALM2 | 3.20 | 5.89 |
| FAM180A | 5.52 | 5.25 |
| STC2 | 5.94 | 4.68 |
| FOXQ1 | 5.01 | 4.28 |
| ADAMTS2 | 5.83 | 4.03 |
| MFAP2 | 2.59 | 3.89 |
| PHLDA1 | 4.72 | 3.86 |
| GRIN2D | 11.31 | 3.81 |
| CDH3 | 5.10 | 3.80 |

Abbreviations: FPKM, Fragments Per Kilobase of exon model per Million mapped fragments; CT, Colorectal tumor; CN, Colorectal normal; FC, Fold change.
