# Supplementary material for: MFAP2, upregulated by m1A methylation, promotes colorectal cancer invasiveness via CLK3
Source: Cancer Med. 2022 Dec 30;12(7):8403–14. doi: 10.1002/cam4.5561 (PMC10134263; doi:10.1002/cam4.5561)
Supplement: Supplementary file 3 — Table S3. [file CAM4-12-8403-s002.docx]

**Supplementary Table S3. Demographic and etiological information of the patients for histological analysis.**

| Variable | Number (175)^#^ | Frequency (%) |
| --- | --- | --- |
| Gender |  |  |
| Male | 91 | 52.0 |
| Female | 84 | 48.0 |
| Age(years) |  |  |
| ≥75 | 41 | 23.4 |
| ≥65, ＜75 | 49 | 28.0 |
| ≥55, ＜65 | 50 | 28.6 |
| ＜55 | 35 | 20.0 |
| T stage |  |  |
| T1 | 1 | 0.6 |
| T2 | 16 | 9.1 |
| T3 | 122 | 69.7 |
| T4 | 36 | 20.6 |
| N stage |  |  |
| N0 | 95 | 54.3 |
| N1 | 47 | 26.9 |
| N2 | 33 | 18.9 |
| M stage |  |  |
| M0 | 146 | 83.4 |
| M1 | 29 | 16.6 |
| AJCC stage |  |  |
| AJCC1 | 16 | 9.1 |
| AJCC2 | 76 | 43.4 |
| AJCC3 | 54 | 30.9 |
| AJCC4 | 29 | 16.6 |

T, Tumor; N, Lymph node; M, Metastasis; AJCC, American Joint Committee on Cancer;

^#^One point of CRC tissue was detached from the slides. As a result, only 174 CRC samples were used to correlate the expression of MFAP2 with clinical characteristics in Table 1.
