# Supplementary material for: MFAP2, upregulated by m1A methylation, promotes colorectal cancer invasiveness via CLK3
Source: Cancer Med. 2022 Dec 30;12(7):8403–14. doi: 10.1002/cam4.5561 (PMC10134263; doi:10.1002/cam4.5561)
Supplement: Supplementary file 4 — Table S4. [file CAM4-12-8403-s003.docx]

**Supplementary Table S4. Altered proteins screened by LC-MS/MS following MFAP2 knockdown in HCT116 cells.**

| **Protein accession** | **Gene name** | **MFAP2 si /Negative si** | **P value** | **Coverage [%]** | **Peptides** | **Unique peptides** | **PSMs** |
| --- | --- | --- | --- | --- | --- | --- | --- |
| Q9H8M2 | BRD9 | 0.425 | 0.0007608 | 2 | 1 | 1 | 1 |
| P08727 | KRT19 | 0.525 | 0.0842723 | 77.5 | 30 | 1 | 1 |
| P22392 | NME2 | 0.540 | 0.0539115 | 48 | 8 | 2 | 26 |
| Q9H0A8 | COMMD4 | 0.544 | 0.0129776 | 9 | 2 | 2 | 3 |
| O43286 | B4GALT5 | 0.635 | 0.0113538 | 4.4 | 1 | 1 | 1 |
| Q8N141 | ZFP82 | 0.649 | 0.0356766 | 5.1 | 2 | 2 | 2 |
| O95297 | MPZL1 | 0.661 | 0.0502845 | 17.5 | 4 | 4 | 5 |
| O95478 | NSA2 | 0.664 | 0.009028 | 24.6 | 6 | 6 | 6 |
| Q92766 | RREB1 | 0.666 | 0.0058163 | 1.7 | 3 | 3 | 3 |
| P99999 | CYCS | 0.674 | 0.0019065 | 47.6 | 4 | 3 | 4 |
| Q6NUS6 | TCTN3 | 0.679 | 0.0796573 | 2.8 | 1 | 1 | 2 |
| Q8WWI5 | SLC44A1 | 0.680 | 0.0052777 | 5.6 | 3 | 3 | 3 |
| Q92871 | PMM1 | 0.687 | 0.0883136 | 11.1 | 2 | 2 | 3 |
| Q8ND82 | ZNF280C | 0.691 | 0.049854 | 7.7 | 6 | 5 | 6 |
| Q9UBI9 | HECA | 0.692 | 0.0139852 | 4.1 | 2 | 2 | 2 |
| Q8N350 | CBARP | 0.693 | 0.0144862 | 2.7 | 2 | 2 | 2 |
| Q8NDX6 | ZNF740 | 0.710 | 0.0277629 | 10.4 | 2 | 2 | 2 |
| Q9NYP7 | ELOVL5 | 0.714 | 0.0010445 | 2.7 | 1 | 1 | 2 |
| Q5SNV9 | C1orf167 | 0.716 | 0.0201102 | 0.7 | 1 | 1 | 1 |
| Q13671 | RIN1 | 0.720 | 0.0437434 | 9.7 | 5 | 5 | 6 |
| Q9HDC5 | JPH1 | 0.723 | 0.0102668 | 10.1 | 6 | 6 | 8 |
| Q96LX8 | ZNF597 | 0.723 | 0.0334535 | 6.8 | 2 | 2 | 2 |
| Q07817 | BCL2L1 | 0.723 | 0.0621381 | 14.2 | 3 | 3 | 4 |
| Q3ZCM7 | TUBB8 | 0.725 | 0.0978051 | 18.9 | 6 | 1 | 39 |
| P09668 | CTSH | 0.727 | 0.0390012 | 3.9 | 1 | 1 | 1 |
| Q9BVK6 | TMED9 | 0.733 | 0.0009378 | 25.1 | 5 | 3 | 11 |
| Q13595 | TRA2A | 0.735 | 0.0069482 | 7.4 | 2 | 2 | 3 |
| Q9UII2 | ATP5IF1 | 0.739 | 0.0231359 | 24.5 | 4 | 4 | 7 |
| P07858 | CTSB | 0.740 | 0.0220402 | 22.7 | 7 | 7 | 10 |
| Q8TF40 | FNIP1 | 0.741 | 0.06492 | 0.7 | 1 | 1 | 1 |
| Q9NX76 | CMTM6 | 0.743 | 0.0218154 | 3.8 | 1 | 1 | 1 |
| O00478 | BTN3A3 | 0.745 | 0.0093803 | 2.2 | 1 | 1 | 1 |
| O95625 | ZBTB11 | 0.746 | 0.0409732 | 6.7 | 6 | 6 | 6 |
| Q9BPW8 | NIPSNAP1 | 0.751 | 0.0232101 | 21.1 | 6 | 4 | 9 |
| P49761 | CLK3 | 0.753 | 0.0807371 | 8.2 | 5 | 5 | 5 |
| O00142 | TK2 | 0.756 | 0.001077 | 6.8 | 2 | 2 | 2 |
| Q96PE2 | ARHGEF17 | 0.757 | 0.018592 | 0.7 | 1 | 1 | 1 |
| Q08357 | SLC20A2 | 0.758 | 0.023215 | 3.8 | 2 | 2 | 2 |
| Q9H8W4 | PLEKHF2 | 0.758 | 0.0229371 | 3.6 | 1 | 1 | 2 |
| Q9H5K3 | POMK | 0.761 | 0.0040208 | 10.6 | 3 | 3 | 4 |
| O75380 | NDUFS6 | 0.761 | 0.044118 | 29 | 4 | 4 | 7 |
| Q9BQE4 | SELENOS | 0.763 | 0.06393 | 24.9 | 4 | 4 | 4 |
| P78364 | PHC1 | 0.766 | 0.040518 | 1.7 | 1 | 1 | 1 |
| P52943 | CRIP2 | 0.767 | 0.056139 | 11.1 | 2 | 2 | 3 |
| O14548 | COX7A2L | 0.769 | 0.0033497 | 27.2 | 3 | 3 | 3 |
| Q15386 | UBE3C | 1.302 | 0.0020081 | 5.3 | 5 | 5 | 6 |
| P09104 | ENO2 | 1.314 | 0.00147 | 28.1 | 9 | 8 | 16 |
| Q96IV0 | NGLY1 | 1.316 | 0.0119268 | 2.1 | 2 | 2 | 2 |
| Q9BYM8 | RBCK1 | 1.328 | 0.0101901 | 4.1 | 2 | 2 | 3 |
| Q96E09 | PABIR1 | 1.339 | 0.0755862 | 9.4 | 1 | 1 | 2 |
| O75674 | TOM1L1 | 1.342 | 0.0745602 | 11.3 | 4 | 4 | 4 |
| A0PK00 | TMEM120B | 1.342 | 0.0041397 | 5 | 1 | 1 | 1 |
| O60732 | MAGEC1 | 1.344 | 0.0952205 | 1.7 | 2 | 2 | 2 |
| Q86U90 | YRDC | 1.351 | 0.0090914 | 9.7 | 2 | 2 | 2 |
| Q02446 | SP4 | 1.362 | 0.0969163 | 6.2 | 3 | 3 | 3 |
| P07951 | TPM2 | 1.364 | 0.0172939 | 31 | 10 | 1 | 1 |
| Q15011 | HERPUD1 | 1.377 | 0.0118616 | 2.3 | 1 | 1 | 1 |
| Q9ULQ0 | STRIP2 | 1.418 | 0.0623397 | 1.9 | 1 | 1 | 1 |
| Q9Y3L3 | SH3BP1 | 1.420 | 0.0196594 | 21.4 | 12 | 1 | 12 |
| Q9BT25 | HAUS8 | 1.425 | 0.0507233 | 10 | 3 | 3 | 3 |
| Q6N043 | ZNF280D | 1.431 | 0.0157046 | 4.2 | 4 | 3 | 4 |
| Q86X95 | CIR1 | 1.462 | 0.0396712 | 5.1 | 3 | 3 | 3 |
| P53779 | MAPK10 | 1.481 | 0.0184069 | 8.8 | 4 | 2 | 6 |
| Q14657 | LAGE3 | 1.481 | 0.0053351 | 21 | 2 | 2 | 3 |
| Q12974 | PTP4A2 | 1.495 | 0.0299445 | 14.4 | 3 | 1 | 3 |
| P17017 | ZNF14 | 1.499 | 0.0124913 | 13.1 | 4 | 1 | 7 |
| P62328 | TMSB4X | 1.511 | 0.0222935 | 25 | 1 | 1 | 1 |
| P15056 | BRAF | 1.536 | 0.0249036 | 6 | 5 | 1 | 6 |
| Q9BQ95 | ECSIT | 1.563 | 0.0501449 | 11.1 | 4 | 4 | 5 |
| Q9P2X3 | IMPACT | 1.582 | 0.025517 | 9.7 | 2 | 2 | 2 |
| A8MTQ0 | NOTO | 1.664 | 0.0781707 | 5.2 | 1 | 1 | 1 |
| O60282 | KIF5C | 1.689 | 0.0023785 | 12.5 | 12 | 2 | 22 |
| P36959 | GMPR | 1.704 | 0.0760676 | 21.4 | 5 | 4 | 9 |
| Q9BY76 | ANGPTL4 | 1.799 | 0.0003597 | 4.7 | 2 | 2 | 2 |
| Q0VDD8 | DNAH14 | 1.866 | 0.0061825 | 0.8 | 2 | 2 | 2 |

Abbreviations: LC-MS/MS, Liquid chromatography tandem mass spectrometry; PSM, Peptide-Spectrum matching.
