# Supplementary material for: MFAP2, upregulated by m1A methylation, promotes colorectal cancer invasiveness via CLK3
Source: Cancer Med. 2022 Dec 30;12(7):8403–14. doi: 10.1002/cam4.5561 (PMC10134263; doi:10.1002/cam4.5561)
Supplement: Supplementary file 5 — Table S5. [file CAM4-12-8403-s006.docx]

**Supplementary Table S5. Potential downstream target genes of MFAP2**

| Gene name | MFAP2 si/ Negative si | p value |
| --- | --- | --- |
| BRD9 | 0.43 | 0.001 |
| MPZL1 | 0.66 | 0.050 |
| RIN1 | 0.72 | 0.044 |
| TMED9 | 0.73 | 0.001 |
| TRA2A | 0.74 | 0.007 |
| CTSB | 0.74 | 0.022 |
| CMTM6 | 0.74 | 0.022 |
| CLK3 | 0.75 | 0.081 |
