# Supplementary material for: MFAP2, upregulated by m1A methylation, promotes colorectal cancer invasiveness via CLK3
Source: Cancer Med. 2022 Dec 30;12(7):8403–14. doi: 10.1002/cam4.5561 (PMC10134263; doi:10.1002/cam4.5561)

Supplementary Figure S1. The staining density of MFAP2 in CRC tissues.
The staining intensity was categorized as follows: 3 (strong positive), 2 (medium positive), 1 (weak positive) and 0 (negative).

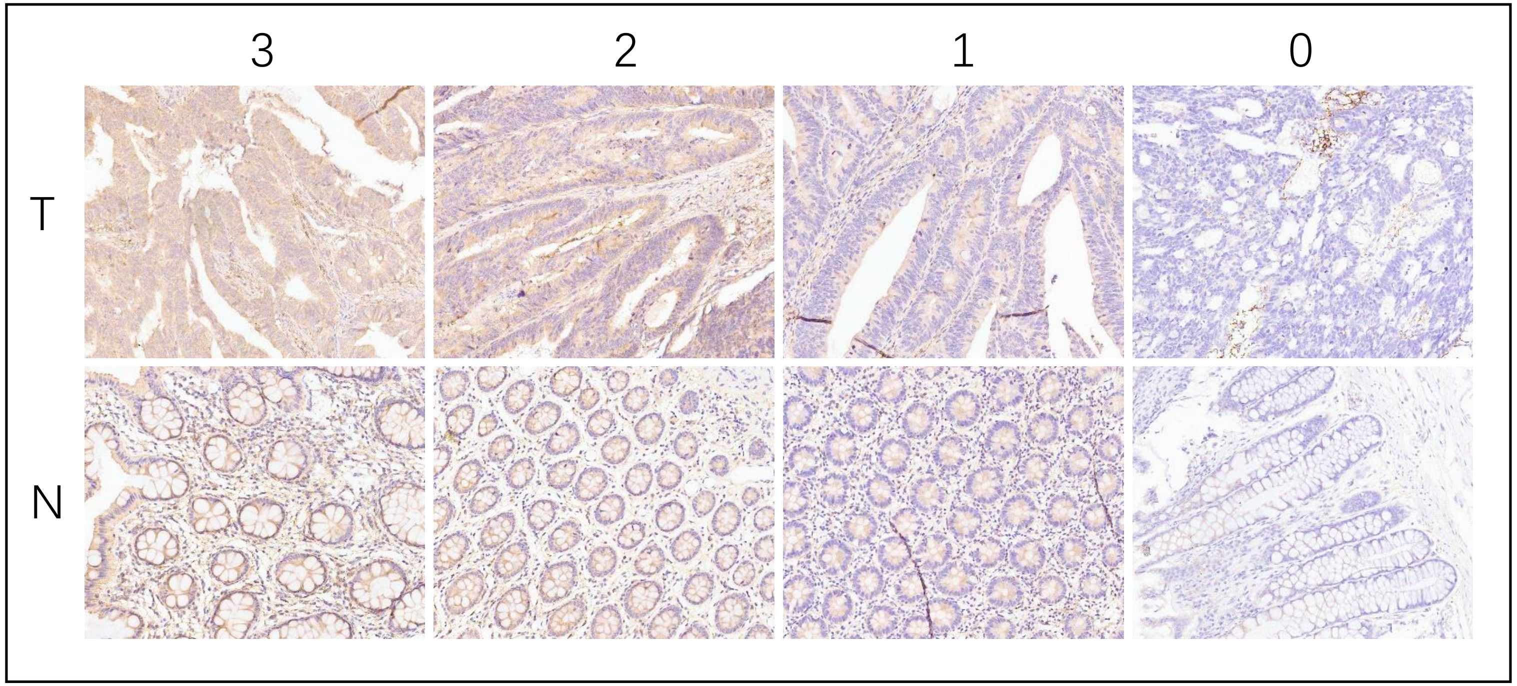

Supplementary Figure S2. ALKBH1 did not affect the m1A modification of LRRC20 mRNA in CRC cells. Relative enrichment of $^{m1A+}$ LRRC20 mRNA (% of Input) in HCT116 and RKO cells after transfecting ALKBH1 siRNA.

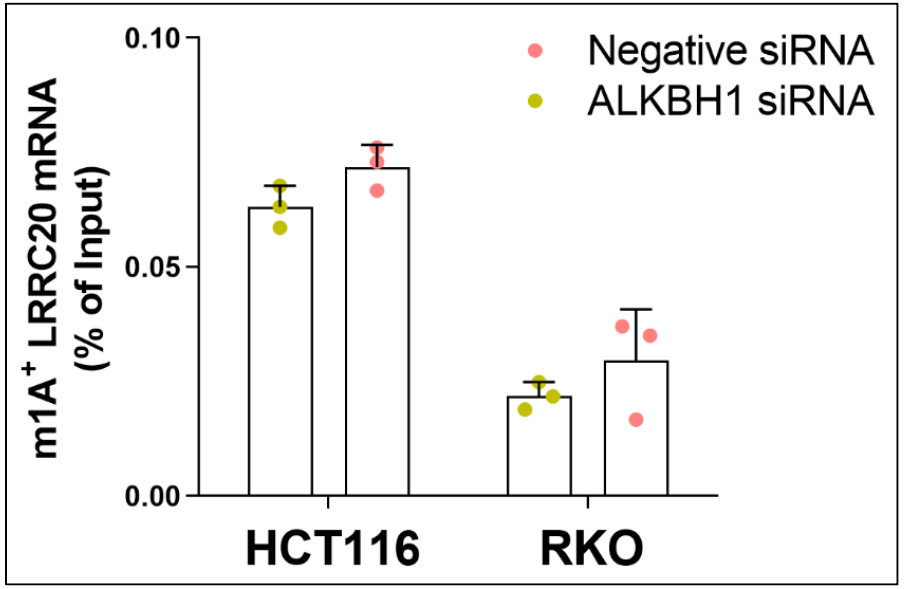

Supplementary Figure S3. Reintroducing ALKBH1 in CRC cells would block the upregulation of $^{m1A+}$MFAP2 mRNA induced by ALKBH1 siRNA. (A) Cotransfection efficiency of ALKBH1 siRNA and ALKBH1 plasmid was validated by Western blot. (B) Relative enrichment of $^{m1A+}$ MFAP2 mRNA in HCT116 and RKO cells after cotransfecting ALKBH1 siRNA and ALKBH1 plasmid (n=3). **p < 0.01 vs Negative siRNA+Empty Vector; $^{\#\#}$p < 0.01 vs ALKBH1 siRNA+Empty Vector.

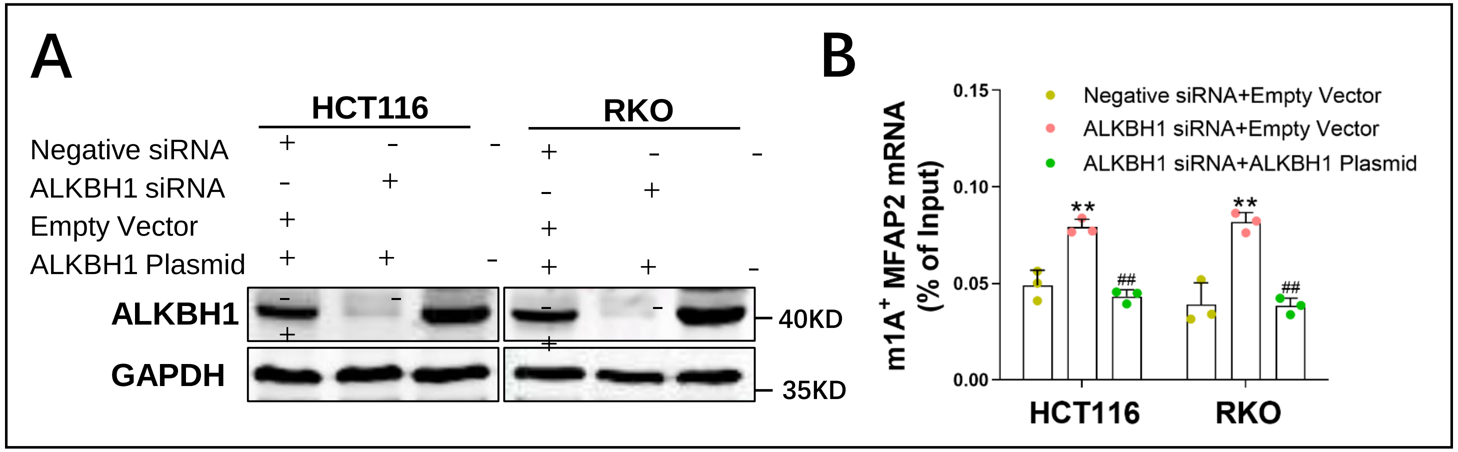

Supplementary Figure S4. Metastatic CRC patients have a higher enrichment of m1A+ MFAP2 mRNA (% of Input) than those non-metastatic CRC patients. Relative enrichment of m1A+ MFAP2 mRNA (% of Input) between T1+T2 and T3+T4 CRC patients (A), N0 and N1+N2 CRC patients (B), M0 and M1 CRC patients (C), AJCC1+2 and AJCC3+4 CRC patients (D). ns, nonsignificant; *$p < 0.05$, vs N0/M0/AJCC1+2.

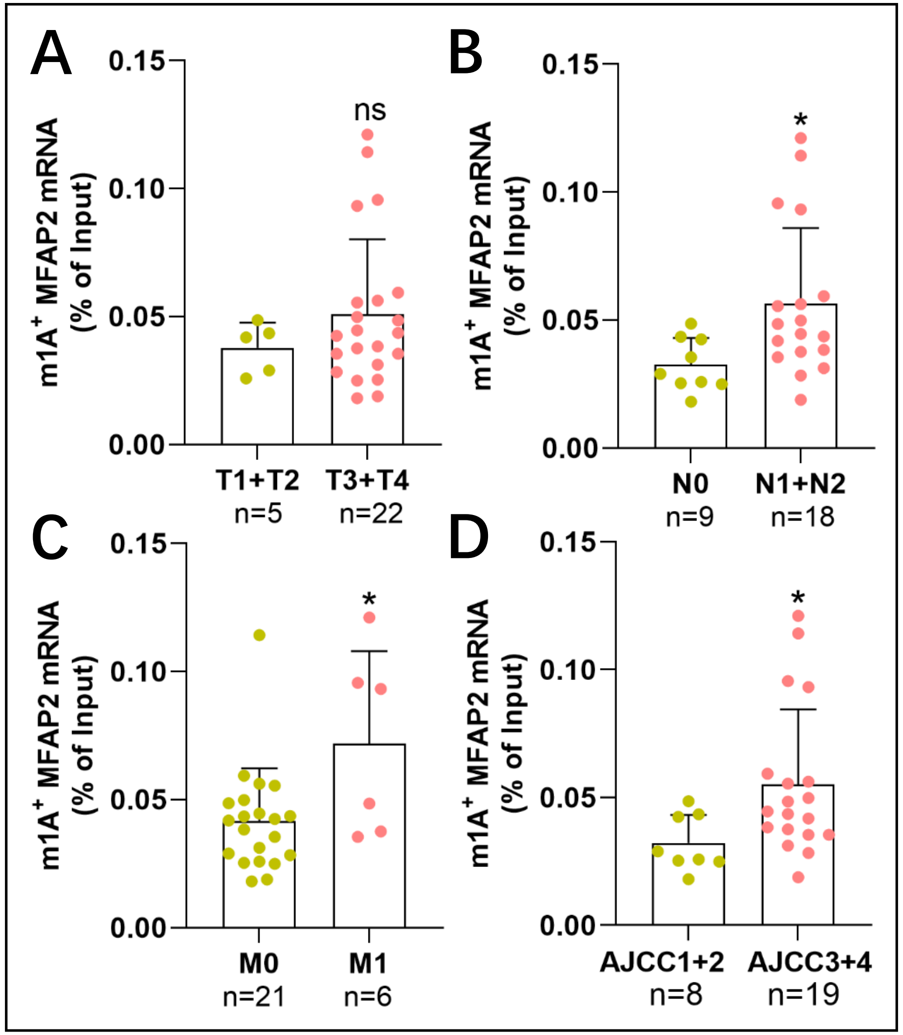

Supplementary Figure S5. Reintroducing MFAP2 would increase the migration and invasion of CRC cells inhibited by MFAP2 depletion. (A) Cotransfection efficiency of MFAP2 siRNA and MFAP2 plasmid was validated by Western blot. (B) After seeded in the upper transwell chamber and incubated for 24 h, HCT116 and RKO cells that migrated to the bottom of the membrane were stained with crystal violet. The representative images were captured under the microscope at 200×. (C) The average number of migratory cells was counted in five random fields (n=5). (D) After seeded in the upper transwell chamber and incubated for 24 h, HCT116 and RKO cells that invaded to the bottom of the membrane were stained with crystal violet. The representative images were captured under the microscope at 200×. (E) The average number of invaded cells was counted in five random fields (n=5). **$p < 0.01$ vs Neg si+Empty Vector; $^{\#\#}p < 0.01$ vs MFAP2 si+Empty Vector; Neg, Negative; si, siRNA.

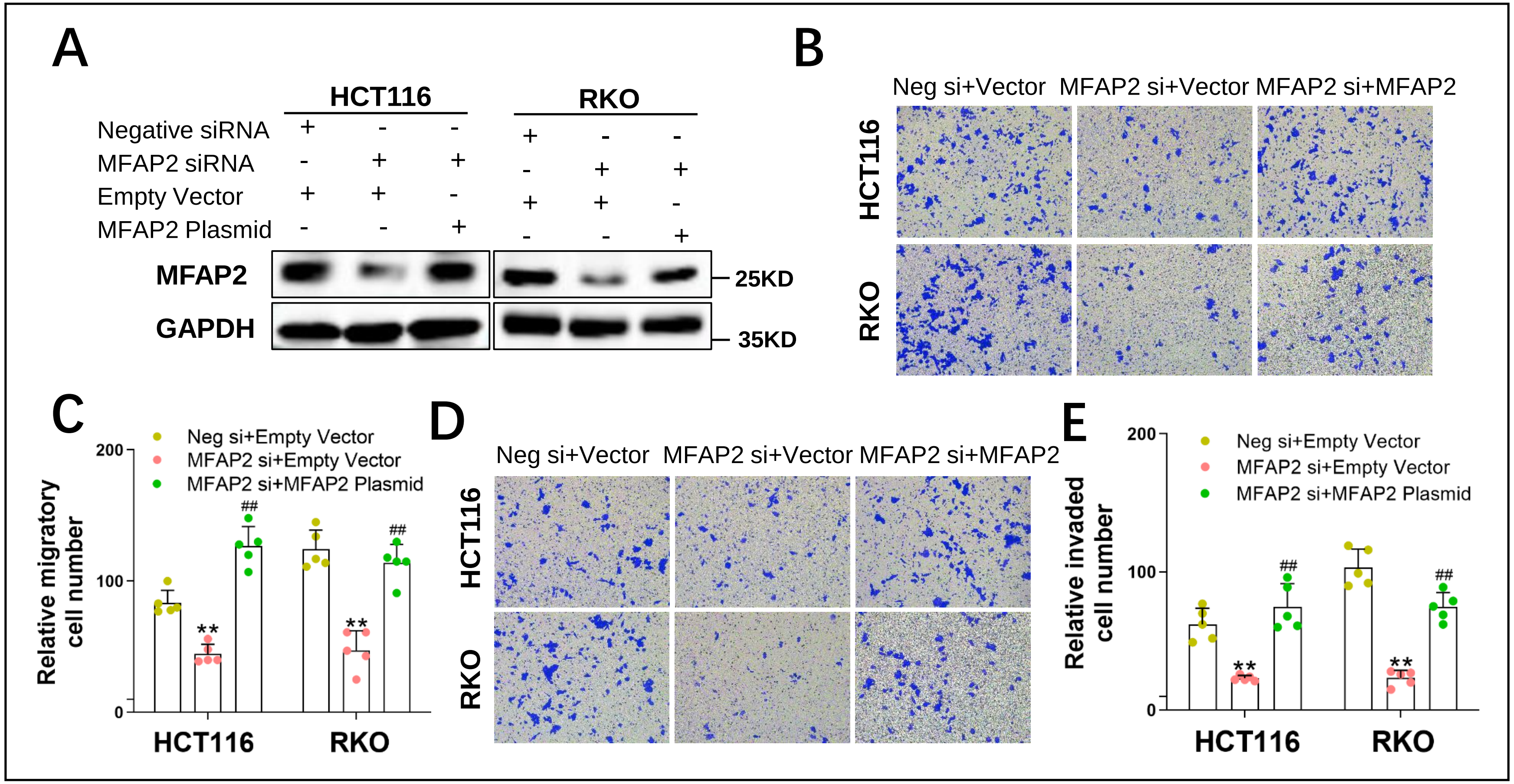

Supplementary Figure S6. Depletion of MFAP2 has no effect on the mRNA expression of CLK3 in CRC cells. The mRNA expression of CLK3 in HCT116 and RKO cells were measured after MFAP2 siRNA was transfected.

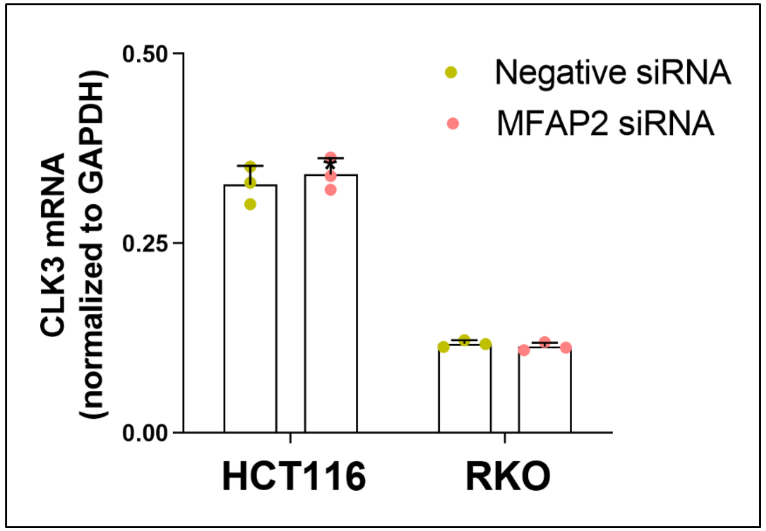

Supplementary Figure S7. The mRNA expression of CLK3 between M0 stage and M1 stage in surgical CRC samples from the GEO dataset of GSE131418.

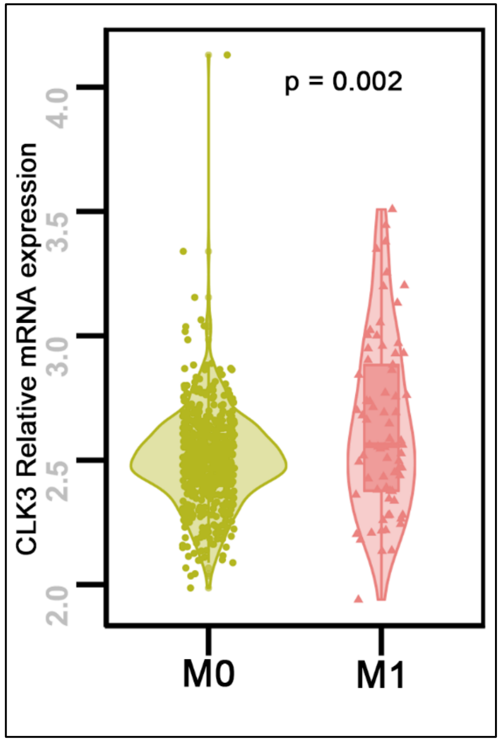

Supplementary Figure S8. Depletion of CLK3 has no effect on the expression of MFAP2 in CRC cells. The mRNA (A) and protein (B) expressions of MFAP2 in HCT116 and RKO cells were measured after CLK3 siRNA was transfected. Neg, Negative; si, siRNA.

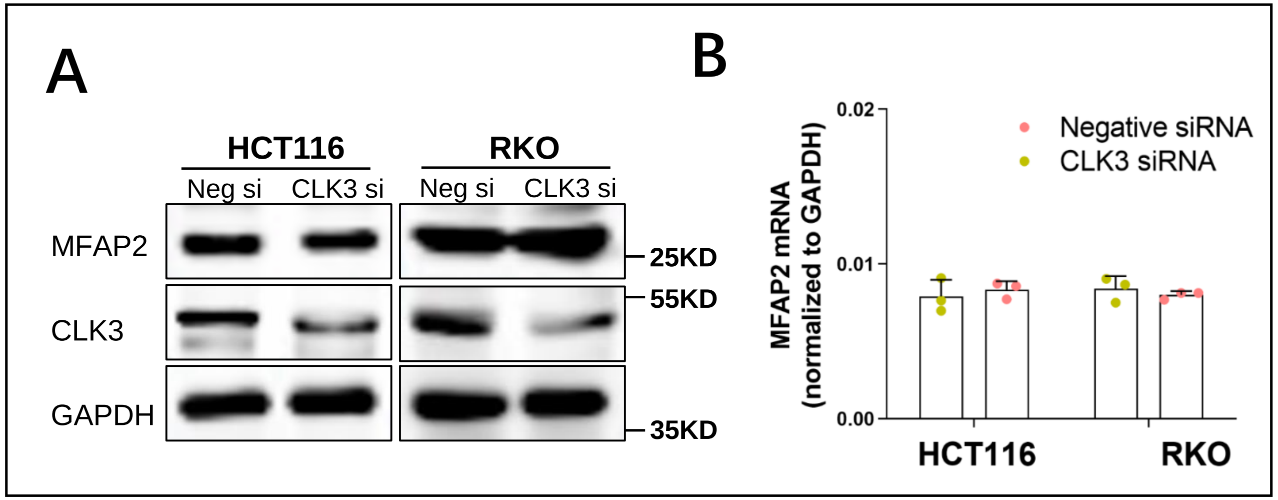

Supplementary Figure S9. Depletion of CLK3 inhibits the migration of CRC cells. (A) After seeded in the upper transwell chamber and incubated for 24 h, HCT116 and RKO cells that migrated to the bottom of the membrane were stained with crystal violet. The representative images were captured under the microscope at 200×. (B) The average number of migratory cells was counted in five random fields (n=5). **p < 0.01 vs Negative siRNA.

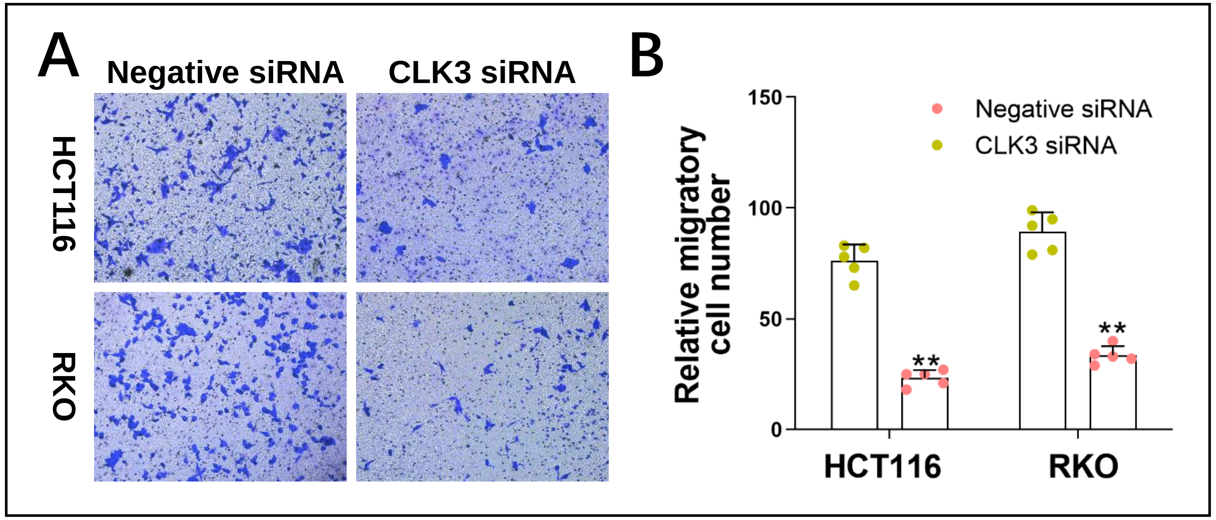

Supplementary Figure S10. MG132 could not rescue the expression of CLK3 inhibited by MFAP2 siRNA in CRC cells. The protein expression of CLK3 was determined by Western blot in MFAP2 silenced HCT116 and RKO cells after MG132 treatment.

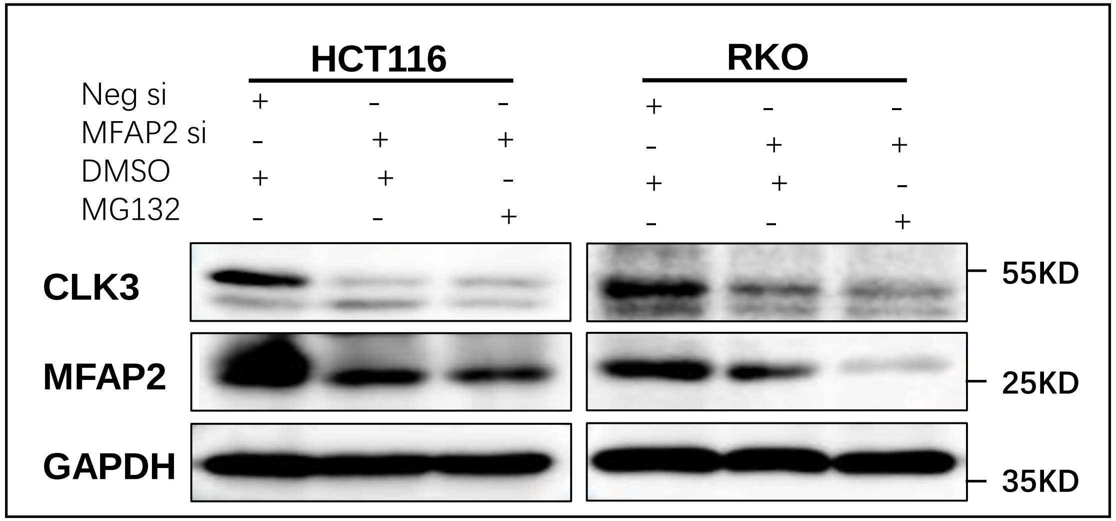

Supplement: Supplementary file 6 — Figure S1–S10. [file CAM4-12-8403-s001.pdf]
